# Distant Non-Obvious Mutations Influence the Activity of a Hyperthermophilic *Pyrococcus* *furiosus* Phosphoglucose Isomerase

**DOI:** 10.3390/biom9060212

**Published:** 2019-05-31

**Authors:** Kalyanasundaram Subramanian, Karolina Mitusińska, John Raedts, Feras Almourfi, Henk-Jan Joosten, Sjon Hendriks, Svetlana E. Sedelnikova, Servé W. M. Kengen, Wilfred R. Hagen, Artur Góra, Vitor A. P. Martins dos Santos, Patrick J. Baker, John van der Oost, Peter J. Schaap

**Affiliations:** 1 Laboratory of Systems and Synthetic Biology, Wageningen University, Stippeneng 4, 6708 WE Wageningen, The Netherlands; vitor.martinsdossantos@wur.nl (V.A.P.M.d.S.); peter.schaap@wur.nl (P.J.S.); 2 Biotechnology Center, Silesian University of Technology, ul. Krzywoustego 8, 44-100 Gliwice, Poland; k.mitusinska@tunnelinggroup.pl; 3 Faculty of Chemistry, Silesian University of Technology, ul. Strzody 9, 44-100 Gliwice, Poland; 4 Laboratory of Microbiology, Wageningen University, Stippeneng 4, 6708 WE Wageningen, The Netherlands; johnraedts@hotmail.com (J.R.); sjon.hendriks@wur.nl (S.H.); serve.kengen@wur.nl (S.V.M.K.); 5 Saudi Human Genome Project, National Center of Genome Technology, King Abdulaziz City for Science and Technology (KACST), Riyadh 11442, Saudi Arabia; almourfif@gmail.com; 6 Bio-Prodict, Nieuwe Marktstraat 54E, 6511 AA Nijmegen, The Netherlands; joosten@bio-prodict.nl; 7 The Krebs Institute for Biomolecular Research, Department of Molecular Biology and Biotechnology, University of Sheffield, Sheffield S10 2TN, UK; s.e.sedelnikova@sheffield.ac.uk (S.E.S.); p.baker@sheffield.ac.uk (P.J.B.); 8 Department of Biotechnology, Delft University of Technology, Van der Maasweg 9, 2629 HZ Delft, The Netherlands; w.r.hagen@tudelft.nl

**Keywords:** Protein engineering, Comulator, cupin phosphoglucose isomerase, *Pyrococcus furiosus*, solvent access

## Abstract

The cupin-type phosphoglucose isomerase (PfPGI) from the hyperthermophilic archaeon *Pyrococcus furiosus* catalyzes the reversible isomerization of glucose-6-phosphate to fructose-6-phosphate. We investigated PfPGI using protein-engineering bioinformatics tools to select functionally-important residues based on correlated mutation analyses. A pair of amino acids in the periphery of PfPGI was found to be the dominant co-evolving mutation. The position of these selected residues was found to be non-obvious to conventional protein engineering methods. We designed a small smart library of variants by substituting the co-evolved pair and screened their biochemical activity, which revealed their functional relevance. Four mutants were further selected from the library for purification, measurement of their specific activity, crystal structure determination, and metal cofactor coordination analysis. Though the mutant structures and metal cofactor coordination were strikingly similar, variations in their activity correlated with their fine-tuned dynamics and solvent access regulation. Alternative, small smart libraries for enzyme optimization are suggested by our approach, which is able to identify non-obvious yet beneficial mutations.

## 1. Introduction

An enormous variety of enzymes can be found in nature, providing a rich source of potential biocatalysts for industrial purposes. However, in the course of natural evolution, these enzymes have been optimized to function optimally in in vivo environments, which may differ substantially from in vitro industrial conditions. Therefore, often there is a need for protein engineering to optimize proteins for their applicability in an industrial setting. Generally, this is achieved by the generation of large libraries of protein variants, from which mutants with improved features are selected. However, the screening of such large libraries is typically costly and time inefficient. Hence, the recently introduced ‘smart library design’ approach, which involves reductions in library size by choosing only a small set of promising amino acid candidates and their substitutions, is considered an appreciable step forward [1,2,3,4,5,6,7]. Smart library design requires the identification of key residues, often through functional predictions using bioinformatics followed by high-throughput experimental analyses. Many such recently-developed bioinformatic approaches can be used to identify promising amino acid candidates, following the semi-rational method, which is a combination of directed evolution and rational design methodologies [1,3,6]. Specifically, the Molecular Class-Specific Information Systems (MCSIS)-based bioinformatics tools, which include the components of (structure-based) large-scale multiple sequence alignments, co-evolution, structure, and automated literature mining, have been shown to be immensely beneficial for a semi-rational method of protein engineering [8,9,10]. An example of such an MCSIS-based bioinformatics approach is our 3DM software suite that contains the standalone ‘Comulator’ tool, which uses a correlated mutation analysis (CMA) algorithm to identify co-evolved residues in large structure-based multiple sequence alignments (MSAs) [11].

Two very distinct roles have been linked to CMA-based residue prediction. On the one hand, correlated residues are proven to represent contact positions in the protein structure, and as such these residues can be used for the prediction of amino acid side chain interactions of the correlated residues [12,13,14]. On the other hand, whenever the correlated residues do not contact each other, and hence are not directly involved in packing within the protein structure [15], co-evolved mutations play a vital role in the protein function [15,16]. Recent studies have shown that the correlated residues in the CMA and their networks of 3DM and other MCSIS-based tools are well suited for the identification of functionally relevant residues [17].

The subject of this study is the cupin superfamily, a large group of structurally-related proteins present in all three domains of life [18]. Members of this superfamily cover a wide range of functions, including isomerases, dioxygenases, oxidoreductases, and storage proteins. The name cupin is derived from the Latin word “cupa” (small barrel), reflecting the conserved beta-barrel structure. High-resolution structures have been obtained for many members of the cupin superfamily [19]. One of the best-characterized members is the phosphoglucose isomerase (PGI) of the archaeon *Pyrococcus furiosus* (PfPGI; EC 5.3.1.9). The aquatic anaerobic hyperthermophilic archaeon *P. furiosus* was first isolated in a near boiling hydrothermal vent [20]. Extensive studies on this hyperthermophile revealed that its optimal growth temperature is 100 °C, and its proteins and enzymes are extremely thermo-stable as well as highly resistant to heat shock and radiation. PfPGI is a glycolytic enzyme that catalyzes the reversible isomerization of glucose-6-phosphate to fructose-6-phosphate (F6P) [21,22]. The bi-directional activity of PfPGI is essential as the enzyme also functions in gluconeogenesis. Several crystal structures of this homodimer (monomeric subunit is 21.5 kDa) have been solved, the coordination of the catalytic metal ion (cofactor) has been elucidated using electron paramagnetic resonance (EPR) analysis, and ample insight into the catalytic mechanism of the enzyme has been gained [23,24,25]. A convergent line of PGI evolution was also elucidated through earlier investigations [26] of the (cofactor) metal-dependent cupin-type phosphoglucose isomerase subfamily and the phosphomannose isomerase (PMI) subfamily of the cupin family. Many structural and combined quantum mechanics/molecular mechanics (QM/MM) studies on PfPGI have also revealed the importance of the indirect and intricate interactions between the cofactor metal ion and critical amino acids (such as His158 and Tyr152) in regulating the potential channel for proton exchange between the substrate and solvent [27]. Moreover, several practical features make PfPGI an ideal candidate for our engineering analysis: Very efficient expression in *Escherichia coli*, straightforward purification and activity assay, and last but not least, the native enzyme is very stable [21].

**Figure 1:**
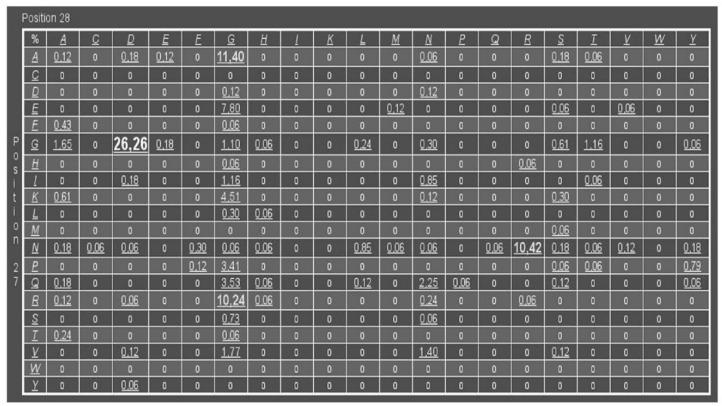
Residue pair frequency table of amino acid couple 27 and 28 (3DM numbering). In wild-type cupin-type *P. furiosus* phosphoglucose isomerase (PfPGI), these residues correspond to Pro132 and Tyr133. The correlated mutation analysis (CMA) scores are shown and are relative to the number of unique sequences containing such occurrences in the superfamily alignment.

In this study, we describe our protein engineering investigations on the hyperthermophilic PfPGI through our combined bioinformatics, biochemical, and biophysical approaches. Our bioinformatics analysis suggested a hot spot located at the periphery of our model enzyme PfPGI (Figure 1) to be the chief co-evolved pair. In our analysis on the complete cupin family CMA, the hot spot residues were (3DM numbers) 27 and 28 in the cupin family, corresponding to Pro132 and Tyr133 in PfPGI, which were highly represented as co-evolving mutations. This hot spot remained non-obvious to other routine protein engineering investigations, which often identify hot spots closer to the binding sites for cofactor and substrate, or involved in the enzyme mechanism or allosteric regulation [28]. Moreover, we could not find any further investigations that followed up on the signature patterns observed in cupin-type PGIs [26]. Since recent studies have highlighted that cold adaptation of enzymes is achieved through conformational changes of regions far from the active site [29,30], we wanted to verify if such changes are also applicable to hyperthermophilic enzymes such as PfPGI. We generated a small ‘smart’ library of variants by randomly substituting the two highly-correlated amino acids based on the suggestions of our bioinformatics tool and performed biochemical screening experiments. We further validated the confirmed relevance of the co-evolved residues by our detailed analysis of selected mutants, including their specific activity, metal coordination, overall 3D structures, molecular dynamics, and a solvent access study. These results show that with regard to designing small ‘smart’ libraries to optimize enzymes through protein engineering, our bioinformatics tool is useful for identifying non-obvious, yet functionally-relevant and beneficial, mutations. However, prudence is recommended during the amino acid candidate selection procedure.

## 2. Materials and Methods

### 2.1. PfPGI-Correlated Mutation Analysis

A refined structure-based MSA of the cupin superfamily, containing a total of 1711 sequences, was used as an input for the Comulator software. The Comulator algorithm derives the CMA scores as described in [31].

### 2.2. Protein and Synthetic Gene Library

Yeast glucose-6-phosphate dehydrogenase was purchased from MP Biomedicals. Chemicals were purchased from Sigma-Aldrich and Roche. The PfPGI mutant library was created by BaseClear (The Netherlands), and the genes were cloned in expression vector pET24d (Novagen).

### 2.3. PfPGI Mutant Library

The cloning of the gene *pgiA* has been described previously [22]. A site-saturation library was designed and created based on CMA using the Comulator software. The constructed library consisted of *pgiA* variants that had alterations in two strongly correlated amino acids: Pro132 and Tyr133. The corresponding numbering in the 3DM alignment was Pro27 and Tyr28. The created *pgiA* mutants were cloned in expression vector pET24d and used to transform *E. coli*.

### 2.4. PfPGI Expression and Purification

Starter cultures of the *pgiA* mutants were inoculated from a glycerol stock and grown in Luria Bertani medium supplemented with 50 μg ml^−1^ kanamycin (LB/Km) in a 37 °C shaker. The overnight culture was used to inoculate (0.2% *v*/*v*) sterile glass tubes containing 10 mL LB/Km medium. When the optical density of the culture reached A600 = 0.5, gene expression was induced by the addition of 0.1 mM isopropyl-1-thio-β-D-galactopyranoside (IPTG). Growth was continued overnight at 37 °C, after which the cells were harvested by centrifugation (4600× *g* for 15 min). Pelleted *E. coli* cells were resuspended in 20 mM Tris-HCl buffer (pH 8.0) and disrupted by sonication. DNase was added to degrade the DNA in the cell lysate to reduce viscosity. Cell debris was removed by centrifugation (16,000× *g* for 15 min). *E. coli* proteins were denatured by heating the cell-free extract at 70 °C for 30 min and removed by centrifugation (16,000× *g* for 15 min). The result was a heat-treated cell-free extract containing mainly PfPGI. Its purity was checked by SDS-PAGE. Protein concentrations were determined by Coomassie Brilliant Blue G250 31, using bovine serum albumin as reference and analysis by SDS-PAGE (Quantity One^®^, Bio-Rad).

PfPGI was purified to homogeneity using an FPLC method similar to that described previously [31]. The heat-treated cell-free extract was diluted to lower the salt concentration, filtered through a 0.45 μm filter and loaded onto a Q-Sepharose Fast Flow column (Amersham Pharmacia Biotech). The column was equilibrated with 20 mM Tris-HCl (pH 8.0). PGI activity eluted at 180 mM of NaCl during a linear gradient of 0 to 1 M NaCl. The fraction with the highest activity was loaded onto a pre-equilibrated Superdex 200 GL column and eluted with 20 mM Tris-HCl (pH 7.0) containing 100 mM NaCl. The protein concentrations and purity were determined, after which the purified enzyme fraction was used for activity assays.

### 2.5. PfPGI Activity Assay

Any divalent metal was stripped from the purified PfPGI using 50 mM EDTA by incubating at 50 °C for 20 min just before the activity measurement. PfPGI activities were determined by measuring NADPH formation in a coupled enzyme assay with yeast glucose-6-phosphate dehydrogenase. This yeast enzyme was present in excess to ensure that the detection of NADPH absorbance at 340 nm (ε = 6.3 mM^−1^ cm^−1^) corresponded to PfPGI activity. The assay mixture contained 0.5 mM NADP, 5 mM F6P, and 0.35 units of D-glucose-6-phosphate dehydrogenase, all in 20 mM Tris-HCl buffer (pH 7.0). All assays were performed using a Hitachi U2001 spectrophotometer with a temperature-controlled cuvette holder set at 50 °C. The optimal activity was measured after careful titration with MnCl_2_, while an excess of this salt resulted in enzyme inhibition.

### 2.6. PfPGI Crystallization

For crystallization, PfPGI was overexpressed and purified as described previously [32]. For each mutant, the protein was concentrated to 11.5 mg mL^−1^ in a solution of 10 mM Tris/HCl, pH 8.0, 50 mM F6P, and 5 mM MnCl_2_. Mutants P132R/Y133G (RG) and P132A/Y133G (AG) were crystallized from hanging drops by mixing equal volumes of protein solution with a reservoir solution containing 0.35 M MgCl_2_, 0.1 M sodium acetate, pH 5.5, and 10–35% PEG4000. For mutants P132A/Y133D (AD) and P132V (VY), crystals were grown using a Hydra Plus One robot and commercial screens. AD was crystallized from a solution of 0.2 M calcium acetate, 0.1 M sodium acetate, pH 6.5, and 40% PEG300; whereas VY was crystallized from a solution of 0.2 M sodium nitrate, 0.1 M Bis Tris Propane, pH 6.5, and 20% PEG4000. A total of 50 mM F6P was added to the AD and VY crystals before mounting. For each different mutant, a single crystal was briefly washed in a cryoprotectant consisting of 25% ethylene glycol in the crystallization buffer, flash cooled to 100 K, and stored in liquid nitrogen before data collection on the Diamond synchrotron light source. Data were processed using Xia2 software [33], and structures determined by molecular replacement using the wild-type PfPGI coordinates as a search model (PDB code: 1X82) [23] and the program Phaser [34]. Rounds of model building using Coot [35] and refinement in Refmac [36] gave acceptable models, verified using MolProbity [37]. For each structure, electron density was present for all the polypeptide chain, and the models had no missing residues. However, the density was weak for the side chains of Lys21, Lys188, and Lys189 (AG); Arg25, Glu114, Asp116, Lys118, Lys188, and Lys189 (RG chain A); Glu114, Lys188, and Lys189 (RG chain B) and Lys188 and Lys189 (AD and VY, chains A and B). Data collection and refinement statistics are given in Supplementary Tables S1 and S2. The four mutant structures were compared to the wild-type Mn/5PAA structure (PDB code: 1X7N) by superposition of all the protein atoms of the residues that coordinate the Mn (His88, His90, Glu97 and His136).

### 2.7. EPR Spectroscopy

Electron paramagnetic resonance spectra were obtained from circa 5 mg ml^−1^ samples of PfPGI mutants in 10 mM Tris-HCl, pH 8.0, to which 0.2 mM of MnCl_2_ was added anaerobically. Spectra were also taken for the PfPGI ternary complexes produced by 10 min incubation with 10 mM F6P. X-band spectra were collected on a Bruker ECS-106 spectrometer using a microwave frequency of 9.45 GHz, a microwave power of 0.126 mW or 126 mW, a modulation frequency of 100 kHz, a modulation amplitude of 6.3 gauss, and a sample temperature of 13 K.

### 2.8. Protein Data Bank Accession Codes

The structure factors and coordinates for the four mutant PfPGI structures have been deposited in the Protein Data Bank with accession IDs: 4LTA (RG), 4LUK (AG), 4LUL (AD), and 4LUM (VY).

### 2.9. Protein Structure Preparation and Molecular Dynamics Simulations

In the AG structure, crystal symmetry relates the two subunits, and; therefore, the two active sites are identical. In the other three structures of the mutants, both active sites are slightly different. Therefore, for all the studied proteins, both wild-type and mutants, the structures of the dimers were prepared for molecular dynamics (MD) simulations. The ligands were manually removed from the active site; only the Mn^2+^ ion was present in the binding cavity. The H++ server [38] was used to protonate each structure using standard parameters and a pH of 7.0. LEaP [39] was used to add counterions, immerse models in a truncated octahedral box of TIP3P water molecules, and to add divalent ion parameters for Particle Mesh Ewald and TIP3P water molecules. The parameters for the Mn^2+^ ion were taken from [40]. Amber 14 [39] was used to run a 100 ns simulation for each analyzed system: the wild-type and the four structures of the mutants. The minimization procedure consisted of 2000 steps, involving 1000 steepest descent steps followed by 1000 steps of conjugate gradient energy minimization, with decreasing constraints on the protein backbone (500, 250, 125, 75, and 25 kcal·mol^−1^·A^−2^) and a final minimization with no constraints of conjugate gradient energy minimization. Gradual heating was performed from 0 to 300 K over 25 ps using a Langevin thermostat with a temperature coupling constant of 1.0 ps in a constant volume periodic box. Equilibration and production were run using the constant pressure periodic boundary conditions for 2 ns with a 1 fs time step and 100 ns with a 2 fs time step, respectively. A constant temperature was maintained using the weak-coupling algorithm for 100 ns of the production simulation time, with a temperature coupling constant of 1.0 ps. Long-range electrostatic interactions were modelled using the Particle Mesh Ewald method with a nonbonded cut-off of 10 Å and the SHAKE algorithm. The coordinates were saved at intervals of 1 ps.

### 2.10. AQUA-DUCT Analysis

AQUA-DUCT software version 0.7.12 [41] was used to trace the paths of all water molecules that were identified during each simulation in each of the analyzed cavities. The analysis of the main cavity was carried out with the object defined as a sphere of 6 Å radius from the center of masses of Val130, Ala135 and the manganese ion, whereas the rear-side cavity analysis was carried out with the object defined as a sphere of 5.5 Å radius from the center of masses of the C-alpha atoms of His90, Leu93, Pro131 and His136. The different radii reflect the size of the cavities detected in the crystal structures. The scope was defined as the interior of the convex hull of C-alpha atoms of each monomer. Each water molecule path was cut to fit to the protein surface (auto_barber set to protein). All inlets were then clustered using the Barber method with cutting sphere correction to the van der Waals radius of the closest atom (auto_barber_tovdw set to True). For the results’ visualization, PyMOL [42] software was used.

AQUA-DUCT was also used for the analysis of the local water molecules’ distribution in the area corresponding to all the paths of all traced molecules. The high-density points or hot spots were located. These are regions where water molecules are either attracted by favorable interactions with nearby amino acids or where they are trapped in hydrophobic cages. In both cases, hot spots can mark regions of particular importance for the enzyme’s functions [43].

## 3. Results and Discussion

### 3.1. DM Analysis of Cupins and Comulator Predictions

**Figure 2:**
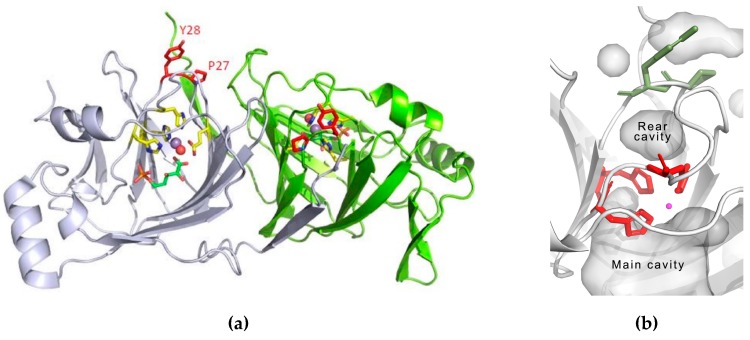
Structure details of wild-type PfPGI. (**a**) Cartoon representation of the dimer (white and green subunits) of the wild-type PfPGI Mn^2+^/5PAA 3D structure (Protein Data Bank (PDB) code: 1X7N). The correlated amino acid pair “PY” is indicated in red. Shown in yellow are those residues involved in manganese (purple sphere) binding, including a water molecule (red sphere). The inhibitor 5-phospho-D-arabinonate (5PAA) is shown as a stick model (green). (**b**) Location of main and rear cavities in PfPGI structure. Histidines 88, 90, and 136 separating main and rear cavities are shown as red sticks, Pro132 and Tyr133 separating rear-side cavity from solvent as green sticks, Mn^2+^ ion as magenta.

A PfPGI library was generated based on the results of the Comulator CMA algorithm, as previously described [31]. We used a refined structure-based MSA of the cupin superfamily, containing a total of 1711 sequences. The amino acid pair with the highest pair-wise correlated mutation score was Pro132 and Tyr133 in PfPGI (3DM-numbers 27 and 28) (Figure 1). This amino acid pair is located in a structurally-conserved peripheral surface loop [31], distant from the catalytic residues and the cofactor binding site, which can be found in most members of the cupin super-family including PfPGI (Figure 2). Previous experiments have shown that a PfPGI double mutant of these two residues exhibited elevated PGI activity levels, while the two single mutants were less active than wild-type PfPGI [31]. These results would not necessarily be predicted, since this peripheral surface loop is not in close proximity to the catalytic residues.

### 3.2. PfPGI Mutant Activity Levels

**Figure 3:**
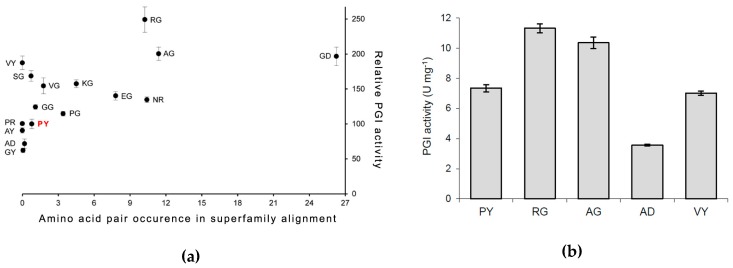
The results of activity measurement: (**a**) Graphical representation of the relative activity of each PfPGI mutant (Y-axis) compared to the amino acid pair occurrence according to the Comulator-based CMA predictions (X-axis). All phosphoglucose isomerase (PGI) activities are relative to wild-type *P. furiosus* PGI (PfPGI; PY; in red). (**b**) The specific activity of wild-type PfPGI (PY) compared to selected high-occurrence/activity mutants P132R/Y133G (RG) and P132A/Y133G (AG), and lower or similar occurrence/activity mutants P132A/Y133D (AD) and P132V (VY). Manganese was used as co-factor and added via titration.

To examine the effect of the correlated residues Pro132 and Tyr133 on PfPGI activity in more detail, we selected 15 mutants (out of the 400 possible) that correspond to amino acid pairs that are either (highly) abundant or (almost) absent within the refined cupin superfamily alignment (Figure 1). Cultures of these mutants could be grown as described previously, and PfPGI expression could be induced successfully for all the mutants. As a control, we included *E. coli* harboring the empty vector (plasmid pET24d), in order to have a correction for background protein concentrations and to exclude possible background activity. Most *E. coli* proteins could be removed from the cell lysate by a heat treatment step and subsequent centrifugation. PfPGI was stripped with EDTA to remove any bound divalent cations and subsequently titrated with Mn^2+^ as the cofactor, as this cation results in the highest in vitro activity [24]. The resulting heat-stable cell-free extract was used for PfPGI activity measurements, to compare the activity of the selected mutants with wild-type PfPGI (Figure 3a).

We could detect PGI activity in the lysates of all the fifteen mutants; none of the PfPGI mutants completely lost activity. The negative control (strain with empty vector) was free of background activity. Hence the measured PGI activity originated only from PfPGI. Significant differences were detected between the specific activities of the examined PGI mutants. Interestingly, we observed elevated activities for those amino acid combinations that, based on the CMA, are abundant in the protein family alignment (for pair frequencies, see Figure 1). For those combinations of amino acids that are absent or less abundant than wild-type PfPGI in the MSA, typical activity levels were observed that were comparable or lower than wild-type PfPGI (amino acid pair PY). These findings suggest a positive correlation between the natural prevalence of an amino acid pair and the activity of the corresponding mutant.

To validate these values, we selected some of these abundantly-expressing mutants for further purification to homogeneity, to enable a more precise analysis of the PfPGI specific activity. In total, we grew five large-batch cultures: wild-type PfPGI (P132/Y133) and mutants P132A/Y133G (AG), P132R/Y133G (RG), P132A/Y133D (AD), and P132V (VY). The first two mutants were chosen because of their high activity in the crude lysates and because their residue pair is highly abundant, while the other two mutants represent an amino acid combination that is either not found in the MSA (VY) or found at a low frequency (AD) compared to the wild-type.

The five PfPGI variants were purified to homogeneity by heat treatment and two subsequent chromatography steps. The resulting pure protein fractions were used to determine the specific activity of each of the PfPGI variants (Figure 3b). When we compared the specific activities of the purified PfPGI variants with those described above for the cell lysates (Figure 3a), similar values were observed. However, the relative activities of the cell lysate fractions were slightly overrated, due to difficulties in obtaining accurate protein concentrations. In comparison with the wild-type PfPGI (PY), we again observed an increased activity for both mutant RG and mutant AG, while both mutant VY and mutant AD had a similar or decreased specific activity, respectively, compared to PY.

The intriguing question to address is the underlying molecular basis of the observed differences in PGI activity. The surface loop carrying the correlated mutations is located somewhat distant from the active site. However, we wanted to examine whether the mutations in the loop have an effect on the active site structure. In addition, we wanted to ascertain whether the mutations had an effect on the delicate solvent channel-based proton exchange between the substrate and solvent that occurs around the manganese metal cofactor [44,45] and the substrate.

### 3.3. Structure Analysis of the Mutation-Carrying Loop

To examine possible conformational changes in the enzyme structure or metal coordination, we initiated crystallization trials for the four PfPGI mutants. First crystallization attempts were set up with manganese as the incorporated cofactor and F6P as substrate. These co-crystallization trials successfully yielded well-diffracting crystals for both the mutants RG and AG.

For the other two mutants (VY and AD), no ternary complex structures were obtained by co-crystallization and thus protein crystals were grown in the presence of MnCl_2_, and subsequently soaked in a solution of F6P for two hours before X-ray data collection. This approach was successful for mutant VY, resulting in a structure with F6P in the active site. For the mutant AD, the only structure that could be obtained contained solely Mn^2+^ in the active site.

The crystal structures of the four mutants revealed that, in each one, clear electron density was present for the 132–133 loop that carries the substitutions. Such clear electron density demonstrates that this loop is not disordered in any of the structures, and that the native fold of PfPGI can accommodate the substitutions within its structure, as inferred from the Comulator predictions. However, as might be expected, a number of differences are noted when comparing the loop structure in detail. In the structure of the wild-type enzyme (P132/Y133), one face of the side chain of Pro132 packs against the carbonyl, Cα and Cβ of Asp94, with the carboxyl of Asp94 pointing away from Pro132. The side chain of Tyr133 packs against the main chain of Leu93, with additional interactions forming between the edge of the phenyl moiety and the side chain of Leu93. The other face of the Pro132–Tyr133 loop packs against the side chains of the Tyr3 residue and the aliphatic part of Lys4 from the second subunit in the PGI dimer. In the structures of the four mutants, the main chain conformation of the 131–134 loop remains remarkably consistent, with only minor changes in the positions of the main chain atoms. However, the changes in the side chains of Pro132 and Tyr133 have more marked effects on the positions of residues 92–94 of the 90–96 loop, and also on the position of the N-terminal five residues from the adjacent subunit of the dimer. There are also some small consequential changes in the positions of second shell residues packing against these two loops.

**Figure 4:**
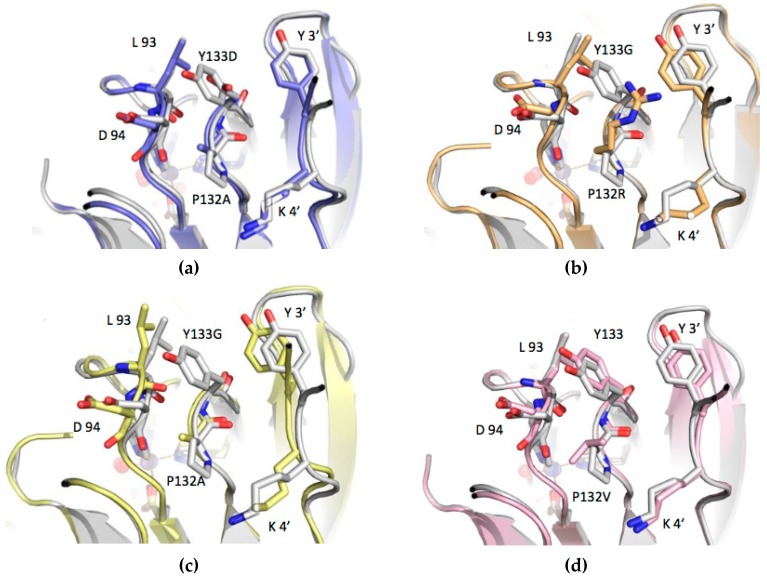
Comparison of the structure of the loops adjacent to the mutation position in the four mutant structures. In each panel, the structure of the wild-type enzyme (PDB code: 1X7N) is shown in white, with the relevant residues highlighted in stick representation and labelled. Y3′ and K4′ refer to the N-terminal strand from the adjacent subunit in the dimer. (**a**) Mutant AD (slate), (PDB code: 4LUL); (**b**) mutant RG (wheat), (PDB code: 4LTA); (**c**) mutant AG (yellow), (PDB code: 4LUK); and (**d**) mutant VY (pink), (PDB code: 4LUM).

For the P132R–Y133G mutant (Figure 4b), the loss of the tyrosine side chain at position 133 is somewhat alleviated by the side chain of R132 occupying approximately the same position in the structure. There are movements of up to 0.5 Å in the positions of both the Leu93–Asp94 and Tyr3–Lys4 loops, compared to the structure of the wild-type enzyme. In the P132V (VY) single mutant (Figure 4d), the change of the proline side chain to valine pushes the Leu93–Asp94 loop away, in order to accommodate the larger bifurcated side chain of valine. Movements of 0.4 and 0.7 Å are seen between the Cαs of Leu93 and Asp94, respectively. In the mutant AD (Figure 4a), the movements of the alpha carbons of Leu93 and Asp94 away from 132–133 are 0.7 and 0.9 Å, respectively. In mutant AD, the change from tyrosine to the negatively-charged aspartic acid has little effect on the position of the side chain of Tyr3 from the adjacent subunit. In the P132A–Y133G mutant (Figure 4c), the changes to the position of Leu93 and Asp94 are more evident, with movements of 1.8 and 1.6 Å for the alpha carbons of Leu93 and Asp94, respectively, compared to the structure of the wild-type enzyme. Given that both mutations in this AG structure are to smaller residues than those in the wild-type, these fairly large movements are, somewhat counter-intuitively, away from the 132–133 pair, presumably making the packing worse. Instead, the 131–134 loop stays packed against the Tyr3 and Leu4 residues from chain B of the homodimer, similar to a lock in all other structures, which is absent in the monomer AG mutant (Figure 4c). The conformation of the loops near the 131–134 loop in all the investigated structures leads to the formation of a rear-side cavity with a different volume, as well as the lining up of a variety of residue side chains around it.

### 3.4. Structure Analysis of the Manganese Coordination

**Figure 5:**
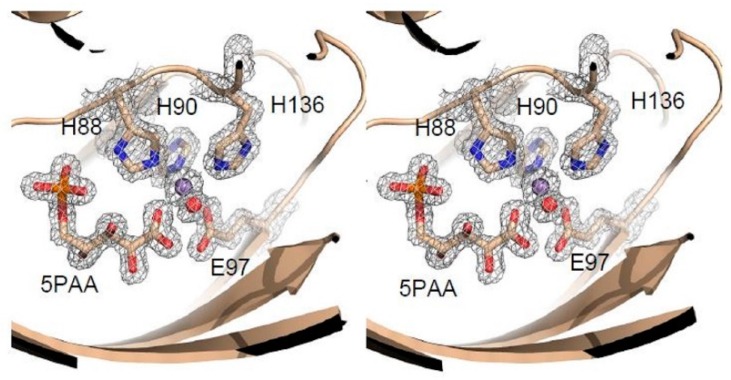
A stereo representation of the mF_O_–DF_C_ electron density (grey mesh), contoured at 1.5σ, for the P132A, Y133G mutant PfPGI structure (mutant AG, PDB code: 4LUK), showing the manganese (purple sphere), coordinating water (red sphere), 5PAA and metal coordinating residues (sticks).

The AG mutant structure has the highest resolution (1.4 Å) for any PfPGI variant, and interestingly, the electron density map for this structure (and also for the RG structure) clearly showed that 5-phosphoarabinonic acid (5PAA), rather than F6P (as added to the crystallization mixture) was bound in the active site (Figure 5). Thus, an unexpected conversion had occurred: During the experiment F6P had been, at least partially, oxidized to 5PAA, resulting in preferential binding for 5PAA in the active site of both mutant structures. To confirm that 5PAA had indeed been produced, a solution of the same composition as the crystallization solution was analyzed by mass spectrometry after being left at room temperature for one week. The spectra contained a small peak of m/e ratio 259, (F6P), but also many other peaks with m/e ratios lower than F6P, including a large peak with m/e ratio of 245 corresponding to 5PAA, clearly indicating that a breakdown of the sugar had taken place. The oxidation of F6P to 5PAA has been observed previously [46], thus we presume a similar reaction occurred in the crystallization solution.

**Figure 6:**
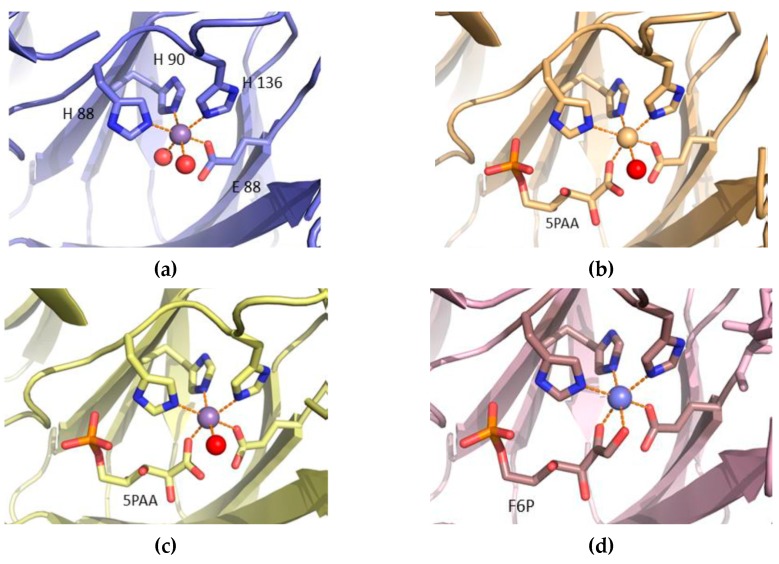
Mn^2+^ coordination in the mutant PfPGI structures. For all mutant structures, the Mn^2+^ ion (purple sphere) is coordinated in an octahedral arrangement: (**a**) Mutant AD (blue), (PDB code: 4LUL); (**b**) mutant RG (wheat), RG (PDB code: 4LTA); (**c**) mutant AG (yellow), (PDB code: 4LUK); and (**d**) mutant VY (pink), (PDB code: 4LUM), respectively. 5PAA and F6P shown as sticks.

In all four PGI mutant structures, the manganese is six-coordinated with an octahedral geometry (Figure 6a–d). Three of the ligands are the imidazole nitrogen of residues His88, His90, and His136. The fourth ligand is one of the carboxyl oxygens of Glu97. The fifth and sixth ligands are oxygen atoms and are different depending on the bound substrate; nevertheless, the relative positions of the coordinating atoms are equivalent. In the mutant AD, water molecules provide these two ligands. In the two structures with 5PAA, mutants RG and AG, the fifth ligand is one of the carboxylate oxygens of 5PAA and the sixth ligand a water molecule. In the F6P-soaked crystal structure of mutant VY, both the fifth and sixth ligands are provided by the F6P substrate; one is the C2 carbonyl and the other the C1 hydroxyl. In this short F6P-soaked crystal structure, there is no indication that any of the F6P has been converted into G6P, as the electron density clearly shows the C2 carbon to have trigonal (sp2) geometry, and the C1 carbonyl to be tetrahedral (sp3), indicating the presence of the ketone isomer of the substrate.

Despite the quite large changes in the relative positions of the 132–133 and 93–94 loops between the different mutants, the position of the manganese coordinating residue His90, which lies only three residues away from the moving residue Leu93, is very similar in all the structures (Supplementary Table S3). The same is true for His88, Glu97 and His136. For a more sensitive investigation of the coordination state of the bound manganese during catalysis, EPR spectral analyses were performed on the four PfPGI mutants, comparing PfPGI without any substrate as well as in a complex with F6P (Supplementary Figure S1). We found that the addition of F6P to any of the manganese-containing PfPGI mutants lead to a collapse of the hexacoordinate manganese signal. Additionally, there is a considerable change in the signal to that of pentacoordinate manganese, leading to a substantial increase in the pentacoordinate to hexacoordinate ratio, indicating a shift towards pentacoordinate metal, the same as that observed for wild-type PfPGI [25]. As the coordination of the Mn^2+^ observed in the crystal structures of the mutants and the dynamic changes in coordination seen in the EPR spectra are both the same as those observed for wild-type PfPGI [25], it is reasonable to conclude that the mutations do not have a large effect on the binding of the metal cofactor.

### 3.5. Molecular Dynamics Calculations and Water Access Analysis

Structural analysis revealed no drastic changes in the conformation and cofactor coordination in the mutants, compared to the wild-type PfPGI. However, it revealed a (rear-side) cavity in all mutants between the highly correlated 132–133 residues and the metal-binding residues His88, His90 and His136. Recent studies on ‘protein sectors’ [47,48] have pointed out that correlated amino acids that are specially located at inter-modular sections could influence activity. Additionally, the dynamics of residues with bulky/aromatic side chains could influence solvent access and subsequently enzyme properties due to their side chain dynamics [49,50]. The water molecules can be even exchange between internal cavities and the bulk solvent by transient tunnels [51]. Since the highly-correlated Pro132-Tyr133 pair and the His88, His90, and His136 residues coordinated to the cofactor Mn^2+^ in PfPGI (along with the His158 and Tyr152 that are involved in the proton transfer) contain such side chains, we considered that their dynamics could similarly vary the solvent access. The presence of water in the rear-side cavity of the mutants also suggested that if the dynamics of these residues (and their side chains) varies, the resulting movement of water through the enzyme will affect activity. Hence, we computed molecular dynamics simulations and solvent access in the four mutants and wild-type PfPGI to understand the effect of mutations on solvent access through them.

The manganese coordination and the presence of water molecules coordinated to it play a role in the activity of PfPGI, as do His158 and Tyr152, which are both involved in the proton transfer and coordinate with the substrate directly or through water molecules [27]. We; thus, instigated a combined molecular dynamics and solvent access analysis similar to our previous investigation [43,50]. For this, we used the dimer structures of the four mutants and the wild-type PfPGI, and changes in each chain were analyzed independently. Careful analysis of the MD simulations of the wild-type PfPGI confirmed the presence of a small rear-side cavity located between the 132–133 residue pair and the His88, His90, and His136 residues, as well as a large active-site cavity (Figure 2b). The small rear-side cavity could accommodate two or three water molecules in it, and the large active-site cavity accommodated substrates. We analyzed in total 500 ns simulations of water flux, which revealed that the main cavities of all variants were permanently open for solvent access with fast exchange of water molecules between the active site cavity and the bulk solvent (Supplementary Table S4).

**Figure 7:**
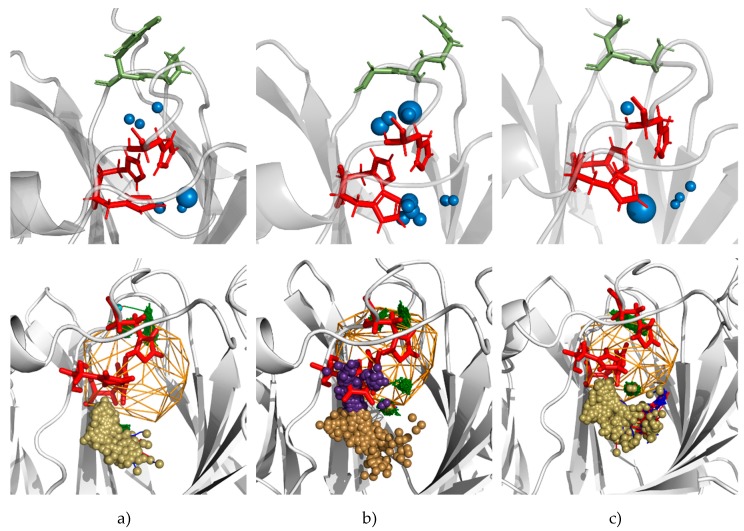
Pictures of water distribution (upper row) and water trajectories (lower row) in the vicinity of the Mn ion in molecular dynamics (MD) simulations of: (**a**) WT chain A (no or small movement of His88, observed in all variants representative for variants with similar to WT activity); (**b**) RG mutant chain B (with His88 rotation observed in chain B of mutants with higher activity), and (**c**) AD chain A (no rotation of His88, lowest water density, observed in mutant with lowest activity). Pictures were generated with PyMOL software based on AQUA-DUCT results. The water distribution pictures present water hot spots as blue spheres with size proportional to the local density of water molecules. Protein backbone—grey cartoon representation; His 88, 90, 136—red stick representation; residues 132 and 133—green stick representation; object—orange mesh; inlets—small balls. Green lines depict trajectories of trapped water molecules. Inlets colors: gold—main cavity cluster, violet—cluster observed due to His88 rotation, cyan—rear-side cavity cluster.

Interestingly, mutations in the 132 and 133 positions in almost all cases had a decreased water flux to the main cavity by the main tunnel (Supplementary Table S1). Only in two cases, RG mutant chain B and AG mutant chain A, was an additional cluster of inlets detected, which was associated with the observed large movements of the side chains of His88 and the loss of the contact between His88 and the Mn^2+^ ion (Figure 7). In the AD and VY mutants, a small His88 rotation was observed; however, ion coordination by all the three histidines was preserved and water entry/exits were distributed in a similar manner to the wild-type (WT), where no movement of His88 was observed. It is worth adding that, for all mutants, the water molecules which were seen in the MD simulations were also trapped in the main cavity, whereas in WT there was no such observation.

Additionally, we inspected the water distribution in the rear-side cavity. During simulations, two or three water molecules were observed in the rear-side cavities in the WT, RG, AG, and VY mutants. In the low activity AD mutant, only one water molecule was present in the rear-side cavity; however, in one chain (exceptionally for a short period) up to three molecules were observed. Concerning water exchange with the bulk solvent, for most of the analyzed variants, the rear-side cavities were separated from the surrounding solvent. Access from the surrounding solvent to the rear-side cavity could probably occur via a bottleneck consisting of Phe89, Leu93, and residue 133; however, excluding the VY mutant, such access was not observed during 100 ns of simulations. These results underline the possible role of the mutants in which the side chains of Pro132 and Tyr133 are substituted in the control of water access. The analysis of water distribution in the main and rear cavities has shown that the local water density in the rear cavity is smallest in the AD mutant, which has the lowest activity (Figure 7).

The main difference that can be observed between the mutants are the differences in accessibility to the active site and rear cavity by water molecules (as seen in the MD simulations) and thus this might be the reason for the differences in activity. We were not able to observe any exchange of water between the rear-side cavity and the main cavity. However, all computational observations suggest that mutations in the loop isolating the rear cavity from the bulk solvent rearrange the local distribution of water molecules and possibly the coordination of the Mn^2+^ ion. The decreased activity of the AD mutant was correlated with the lowest density of water molecules in the rear cavity.

## 4. Conclusions

Our 3DM-based bioinformatics approach and the promising results reiterate the potential use of Comulator-based CMA predictions to identify ‘hot spots’ for designing small ‘smart’ libraries to engineer protein performance. The analysis of crystal structures and EPR experiments has shown that there is no direct evidence that could provide an explanation of the differences in activity. It is possible to conclude that the observed changes in the variants’ performance are caused by subtle structural changes introduced by distant mutations that are propagated throughout the structure. However, using our water tracking approach, we found that the subtle changes in the structure and dynamics of the amino acid side chains might have influenced solvent access regulation in the vicinity of the Mn^2+^ ion, and they can provide an alternative explanation of the observed differences in the mutants’ activity. Since this specific hot spot has a critical effect in inferring functional information, we believe that prudence is required in the selection of correlated resides for such structures and function prediction tools.

## Supplementary Materials

The following are available online at https://www.mdpi.com/2218-273X/9/6/212/s1, Table S1: Crystallography data—collection statistics, Table S2: Crystallography data—refinement statistics, Table S3: Mn2+ ligands and coordination distances (Å), Table S4: Average time required for the exchange of water molecules between the main cavity and bulks solvent for the wild-type and four mutant structures, Figure S1: Invariance of EPR spectra from the PfPGI mutants.
