# Supplementary material for: Distant Non-Obvious Mutations Influence the Activity of a Hyperthermophilic Pyrococcus furiosus Phosphoglucose Isomerase
Source: Biomolecules. 2019 May 31;9(6):212. doi: 10.3390/biom9060212 (PMC6627849; doi:10.3390/biom9060212)
Supplement: Supplementary file 1 [file biomolecules-09-00212-s001.zip › S1.pdf]

Table 1 - Crystallography data—collection statistics

**Data set**	**AG**	**RG**	**AD**	**VY**
Spacegroup	C2	$P2_12_12_1$	$P2_12_12_1$	P1
Unit cell parameters (Å)				
a	87.9	73.0	46.2	80.7
b	43.3	74.5	46.2	42.6
c	58.5	75.9	186.2	88.9
α	90.0°	90.0°	90.0°	90.0°
β	122.0°	90.0°	90.0°	104.9°
γ	90.0°	90.0°	120.0°	90.0°
Molecules per ASU	1	2	2	2
Resolution (Å)[1]	28.92-1.41 (1.44 - 1.41)	26.58-2.04 (2.09 -2.04)	25.58-1.89 (1.94 -1.89)	18.49-1.79 (1.84-1.79)
Wavelength (Å)	0.97630	0.97630	0.97630	0.97630
Unique observations[1]	32797 (1460)	26994 (1969)	29381 (2144)	33074 (2386)
R_{pim}[1]	0.036 (0.349)	0.074 (0.219)	0.028 (0.352)	0.086 (0.349)
Completeness (%)[1]	91.2 (55.4)	99.6 (99.9)	98.5 (99.2)	95.7 (94.0)
Multiplicity[1]	3.1 (2.4)	6.3 (6.4)	3.5 (3.5)	1.8 (1.8)
Mean((I)/sd(I))	13.0 (2.1)	7.0 (3.7)	15.9 (2.4)	4.3 (2.2)

[1] Numbers in parentheses indicate values for the highest resolution shell
