# Supplementary material for: Distant Non-Obvious Mutations Influence the Activity of a Hyperthermophilic Pyrococcus furiosus Phosphoglucose Isomerase
Source: Biomolecules. 2019 May 31;9(6):212. doi: 10.3390/biom9060212 (PMC6627849; doi:10.3390/biom9060212)
Supplement: Supplementary file 1 [file biomolecules-09-00212-s001.zip › S2.pdf]

**Model**	**AG**	**RG**	**AD**	**VY**
Resolution (Å)	1.4	2.0	1.9	1.8
Number of reflections	31134	25635	27836	31398
Protein molecules per asymmetric unit	1	2	2	2
Number of atoms	1739	3221	3142	3308
Number of waters	197	141	112	218
Number of Mn^{2+} ions	1	2	2	2
Number of F6P	0	0	0	2
Number of 5PAA	1	2	0	0
Ramachandran favoured (%)	98.4	87.1	97.3	97.9
Ramachandran outliers (%)	0	0.3	0.3	0
Poor rotamers (%)	1.3	1.6	0.6	0.3
RMSD bond (Å)	0.006	0.007	0.011	0.009
RMSD angle (^{O})	1.06	1.08	1.34	1.26
Average B-factors (Å)				
Main chain	21	35	31	24
Side chain	31	24	34	27
Waters	37	36	33	26
Mn^{2+}	13	27	31	21
5PAA/F6P	15	33	-	26
R-factor	0.14	0.22	0.19	0.17
R-Free	0.20	0.29	0.25	0.22
MolProbity score	0.80 100th percentile	0.94 100th percentile	0.99 100th percentile*	1.03 100th percentile
