# Supplementary material for: Distant Non-Obvious Mutations Influence the Activity of a Hyperthermophilic Pyrococcus furiosus Phosphoglucose Isomerase
Source: Biomolecules. 2019 May 31;9(6):212. doi: 10.3390/biom9060212 (PMC6627849; doi:10.3390/biom9060212)
Supplement: Supplementary file 1 [file biomolecules-09-00212-s001.zip › S3.pdf]

Mutant	His 88-N2	His 90-N2	His 136-N2	Glu 97-
PY WT (1X81)	2.45	2.25	2.29	2.26- O2
RG	2.20	2.30	2.29	2.17-O1
AG	2.32	2.25	2.23	2.33-O1
AD	2.24	2.21	2.35	1.98-O1
VY	2.31	2.31	2.28	2.09-O1
