# Supplementary material for: Distant Non-Obvious Mutations Influence the Activity of a Hyperthermophilic Pyrococcus furiosus Phosphoglucose Isomerase
Source: Biomolecules. 2019 May 31;9(6):212. doi: 10.3390/biom9060212 (PMC6627849; doi:10.3390/biom9060212)
Supplement: Supplementary file 1 [file biomolecules-09-00212-s001.zip › S4.pdf]

Structure	Avg IN [ns]	Avg OBJ [ns]	Avg OUT [ns]
WT (PY) chain A	<0.1 ± <0.1	<0.1 ± 0.1	<0.1 ± <0.1
WT (PY) chain B	<0.1 ± <0.1	0.1 ± 0.1	<0.1 ± 0.1
RG chain A	0.2 ± 0.2	0.2 ± 0.3	0.3 ± 0.2
RG chain B	0.2 ± 1.6	0.1 ± 0.3	0.1 ± 0.3
AD chain A	0.1 ± 0.3	0.2 ± 0.4	0.1 ± 0.3
AD chain B	0.1 ± 0.2	0.1 ± 0.2	0.1 ± 0.1
AG chain A	0.1 ± 0.1	0.1 ± 0.2	0.1 ± 0.1
AG chain B	0.2 ± 0.4	0.2 ± 0.3	0.2 ± 0.2
VY chain A	0.1 ± 0.1	0.1 ± 0.1	0.1 ± 0.1
VY chain B	0.2 ± 0.2	0.2 ± 0.3	0.2 ± 0.2
