# Supplementary figures and images for: Distant Non-Obvious Mutations Influence the Activity of a Hyperthermophilic Pyrococcus furiosus Phosphoglucose Isomerase
Source: Biomolecules. 2019 May 31;9(6):212. doi: 10.3390/biom9060212 (PMC6627849; doi:10.3390/biom9060212)

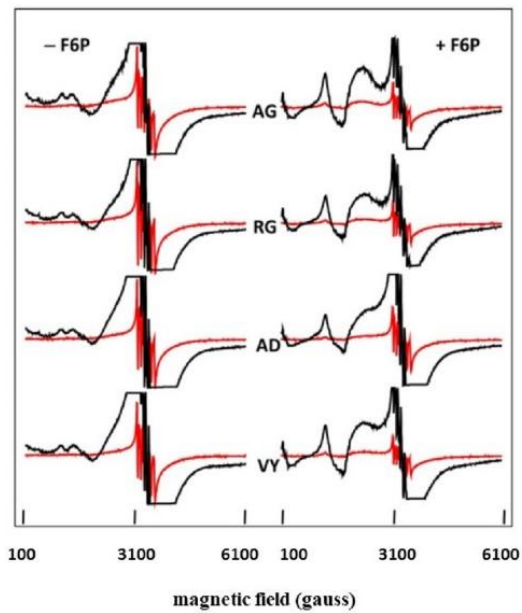

Supplement: Supplementary file 1 [file biomolecules-09-00212-s001.zip › SF1.pdf]
