# Supplementary material for: Distant Non-Obvious Mutations Influence the Activity of a Hyperthermophilic Pyrococcus furiosus Phosphoglucose Isomerase
Source: Biomolecules. 2019 May 31;9(6):212. doi: 10.3390/biom9060212 (PMC6627849; doi:10.3390/biom9060212)
Supplement: Supplementary file 1 [file biomolecules-09-00212-s001.zip › Supplementary captions.doc]

Supplementary captions

Table S1: Crystallography data – collection statistics,

Table S2: Crystallography data – refinement statistics,

Table S3: Mn^2+^ ligands and coordination distances (Å)

Table S4: Average time required for the exchange of water molecules between the main cavity and bulks solvent for the wild type and four mutant structures, Avg IN – average time of trajectories of water molecules traveling to the active site, Avg OUT – average time of trajectories of water molecules traveling from the active site. For comparison, the average time of water trajectories in the main cavity is also provided (Avg OBJ).

Figure S1: Invariance of EPR spectra from the PfPGI mutants. The red traces were taken at low microwave power (0.126 mW) for a full record of the six-line pattern around 3300 gauss typical for hexacoordinate Mn^2+^. The black traces were taken at high microwave power (126 mW) to emphasise the broad features over the whole magnetic-field range from pentacoordinate Mn^2+^. The left-hand traces are in the absence of substrate, while the right-hand traces are in the presence of 10 mM F6P.
